# Human response to live plague vaccine EV, Almaty region, Kazakhstan, 2014-2015

**DOI:** 10.1371/journal.pone.0218366

**Published:** 2019-06-14

**Authors:** Zaurbek Sagiyev, Almas Berdibekov, Tatyana Bolger, Almagul Merekenova, Svetlana Ashirova, Zamir Nurgozhin, Zhandos Dalibayev

**Affiliations:** 1 M. Aikimbayev’s Kazakh Scientific Centre for Quarantine and Zoonotic Diseases, Almaty, Kazakhstan; 2 Taldyqorgan Anti-Plague Station, Taldyqorgan, Kazakhstan; 3 Panfilov Regional Department of Public Health Protection, Zharkent, Kazakhstan; 4 Panfilov Regional National Center of Expertise, Zharkent, Kazakhstan; New York Medical College, UNITED STATES

## Abstract

**Background:**

In Kazakhstan, a live plague vaccine EV 76 NIIEG has been used for plague prophylaxis since the mid-1930s. Vaccination is administered yearly among people living in plague-enzootic areas. Similar practices are used in other former Soviet Union countries. Yet, to this day, the effectiveness period of the vaccine is unknown. It is also not clear how different factors can affect the effectiveness of the vaccine over time.

**Methods:**

We surveyed changes in antibody levels specific for F1 antigens of *Yersinia pestis* among vaccinated people 4, 8, and 12 months post- vaccination. Blood samples were taken from the participants of the study for producing sera, which was later analyzed using indirect hemagglutination reaction with antigenic erythrocyte assay (micromethod) for identifying antibodies to F1 *Y*.*pestis*.

**Results:**

In first-time-receivers of the plague vaccine, antibody titer reached the highest level of antibody that represents a conditionally protective titer after 4 months, dropped drastically after 8 months, and dropped again after 12 months. Similar results were obtained among those who have been vaccinated previously. However, in that group, the percentage of people with a level of antibody that represents a conditionally protective titer remained statistically significant even after 8 and 12 months.

**Conclusion:**

Based on the results of this study, we recommend initiating vaccination campaigns for the medical and veterinary staff, as well as the general population four months prior to the springtime epizootics of plague among wild rodents.

## Introduction

Since plague has taken many human lives in the past. It is not surprising that one of the first attempts to create a vaccine was an attempt to develop a vaccine against the plague. The first effective plague vaccine was created by The Institut Pasteur in late 1890s. It was a killed vaccine. After that, live attenuated vaccines were developed [1]. Currently, two types of plague vaccines are used globally: killed-whole cell (KWC) and live whole-cell (LWC). KWC vaccines use virulent strains killed by heating or adding formaldehyde, whereas LWC vaccines are created on the basis of virulent *Y*. *pestis* strains [2, 3, 4, 5,6].

The plague strain isolated by G. Girard and J. Robic in Madagascar from a girl who died of a bubonic plague was used for the LWC vaccine. LWC vaccine based on attenuated strain of *Y*. *pestis* (EV76) was created at the Scientific Research Institute of Epidemiology and Hygiene (Russian abbreviation—NIIEG, Kirov) in 1936. It was used for vaccinating the laboratory staff and the population of plague enzootic areas [5]. This vaccine is licensed for use against plague and is still being used in former French colonies, Mongolia, and China [7]. It was introduced in the Soviet Union in the mid-1930s and is being used in Post-Soviet countries to this day [8–10].

In the last 80 years, millions of people were vaccinated using LWC. Not a single case of serious side effects or diseases caused by the vaccine was registered during that time [8]. The side effects, when they are present, are usually minor and do not last long. It is believed that the vaccine remains effective for a year after its administration [8]. LWC protects both against the bubonic and pneumonic plague [5]. Vaccination is performed once percutaneously. Revaccination is performed after 12 months. LWC is considered highly reactive and is not licensed in Europe and the USA [9,10].

KWC was developed in the US in 1946 for vaccination of army personnel [3, 10]. It was first produced by Cutter Laboratories (USA) and later by Geer Laboratories (USA) on the basis of 195/P strain of *Y*.*pestis* killed by adding formaldehyde [3, 10]. Currently, an alternative plague vaccine containing plague strains killed by heating is produced by Commonwealth Serum Laboratories in Australia [10]. The vaccine is injected into human body intramuscularly in three stages with intervals of 1 to 3 months. The third dosage is administered 6 months after the first and second ones. Revaccination is done after 1–2 years. People living in endemic areas and laboratory staff are subject to vaccination [3]. KWC is effective against bubonic plague but weak against pulmonary plague. The vaccine also requires multiple immunizations [10]. Compared to EV76 it is less effective [4, 11].

Specific IgG subclasses to the capsular antigen F1 are considered markers of immunity in people vaccinated against plague. At the same time, it is still unknown for how long those antibodies are present in vaccinated people. There was not a single randomized clinical study aimed at answering that question [10].

This prospective study aims to determine the effectiveness period of serum antibodies against Fraction 1 of *Y*. *pestis* in people vaccinated with LWC vaccine EV 76 NIIEG. Specifically, it aims to answer the following two questions: (1) how do antibody levels change after 4, 8, and 12 months, and (2) how do factors, such as previous plague vaccination history, age, gender, occupation (medical specialist/non-medical specialist), rodent exposure, and level of education affect the period of effectiveness. We added these factors (education, occupation) because among the vaccinated people there were microbiologists, biologists who worked in the field with wild plague foci elements (mammals, insects, etc.) and could contact with the biological objects infected with the plague that could theoretically change the level of antibodies.

## Materials and methods

493 people vaccinated with LWC vaccine EV 76 NIIEG, ages 20 to 69, participated in the study, including 243 men and 250 women. 352 were vaccinated against plague 1–4 times, while 141 people have received vaccine 5 or more times. The vaccinated people worked in different institutions (Table 1).

**Table 1:** People participated in the study.

Variables		Number of people before vaccination	Number of people in 4 months after vaccination	Number of people in 8 months after vaccination	Number of people in 12 months after vaccination
Age groups	40+ years	276	256	245	251
	< 40 years	217	198	196	202
Education group	14+ years	137	124	121	126
	< 14 years	356	330	320	327
Sex	Male	243	225	214	220
	Femals	250	229	227	233
Work place	Veterinary Stations	77	64	65	69
	Anti-plague Stations	139	135	121	121
	General population	277	255	255	263
Vaccination frequency	5+ vaccinations	141	135	128	129
	1–4 vaccinations	161	140	146	150
	0 vaccinations before 2014	191	179	176	174
Contacts with rodents	yes	59	58	51	51
	no	433	395	389	401

**Fig 1:**
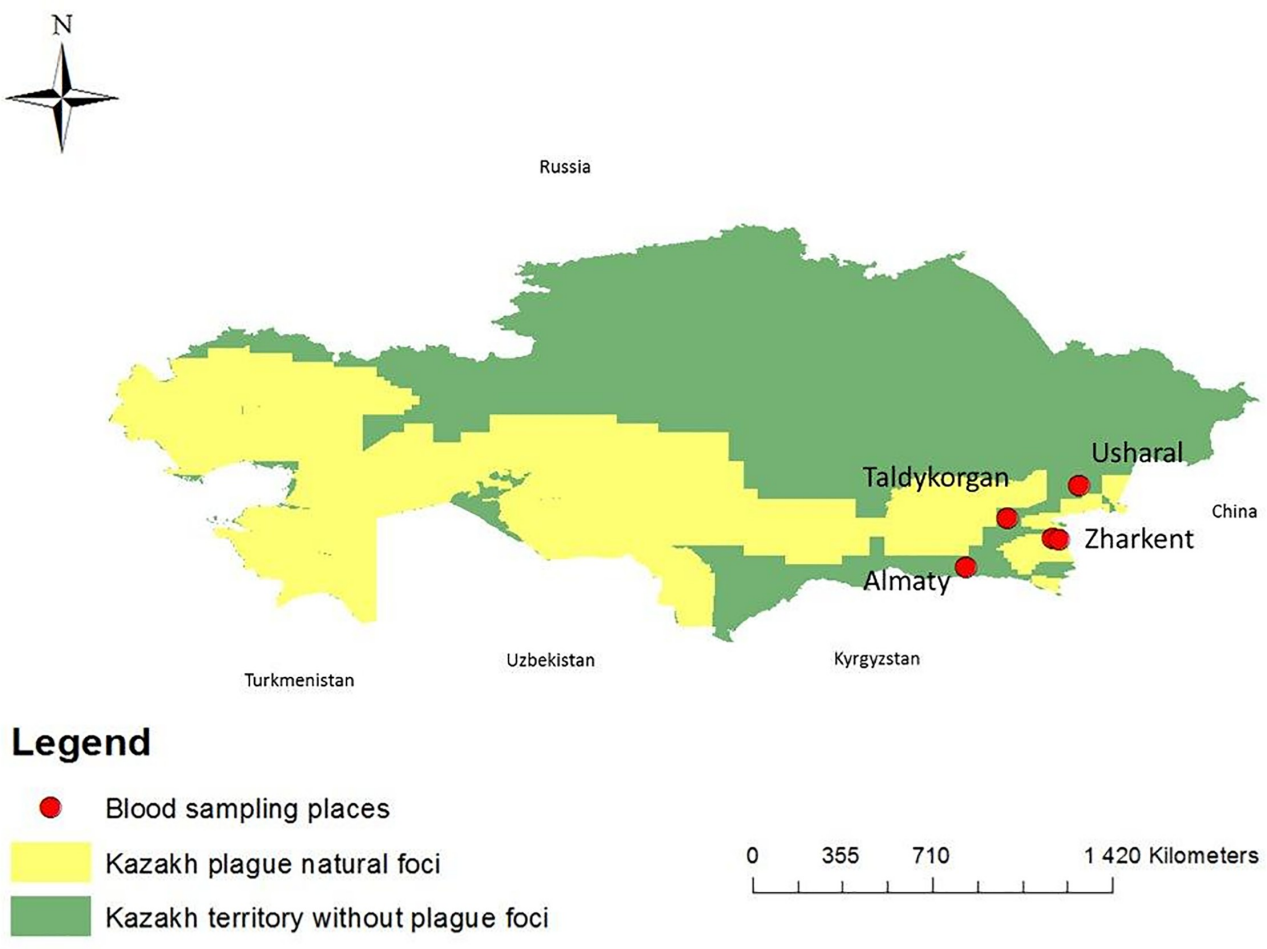
Natural plague foci in Kazakhstan and places where blood samples from vaccinated people were collected.

The vaccination was administered percutaneously once. We measured F1 *Y*.*pestis* antibody levels in participants of the study before vaccination and 4, 8, and 12 months post-vaccination. The number of participants varied throughout the year. All 493 were present for the first blood sampling before the vaccination, 454 were present for the second blood sampling (four months after the vaccination), 441 were present for the third blood sampling (eight months after the vaccination), and 453 were present for the fourth blood sampling (12 months after the vaccination) (Table 1). Moving to a different location, changing jobs, and vacation were cited among reasons for not attending a scheduled blood sampling (Fig 1).

Blood samples were taken from the participants of the study for producing sera, which was later analyzed using indirect hemagglutination reaction with antigenic erythrocyte assay (micromethod) for identifying antibodies to F1 *Y*.*pestis* [9]. Whole serum was first diluted 1:10 with 0,9% saline solution pH 7,2, then inactivated by heating to 56^0^С in a water bath, and finally treated with 50% suspension of formalized erythrocytes in order to neutralize heterogeneous antibodies for sheep erythrocytes. Antigenic erythrocyte plague assay, series 010314, К№ 262, produced by KSCQZD was used for the study. Agglutinating serum for plague with the titer of 1:10, series #010214, КN 264, was used for controls. We used the titer of 1:160 as cutoff for a positive antibody response against F1 antigen [12].

Epi Info, version 7 [13] was used for data analysis. The immune answers before and after the vaccination were compared using McNemar’s test. P<0.05 was considered a significant result. Odds ratio was used to determine whether a specific factor affected the level of antibodies (OR = 1 exposure did not affect odds of outcome, OR>1 Exposure associated with higher odds of outcome, and, OR<1 Exposure associated with lower odds of outcome). Multivariate regression analysis was used for testing associations between positive immune answers and the proposed variables: previous plague vaccination history, age, gender, occupation (medical specialist/non-medical specialist), rodent exposure, and level of education. For previous plague vaccination history, the values were: those who have been vaccinated in the past and those who have not. For age, the variables were under 40 and over 40. For gender, the variables were male and female. For occupation, the variables included medical/veterinary specialists and non-medical/veterinary specialists. For rodent exposure, the variables were ‘had exposure’ and ‘did not have exposure’. For the level of education, the variables included higher education (14 years or more) and secondary education (less than 14 years). We chose all variables based on our discussing with local Public Health Authorities (Table 1).

The study was approved by the Local Ethics committee of the Kazakh National Medical University named after S.D. Asfendiyarov.

## Results

In Kazakhstan, the plague enzootic area is 1,007,350 km^2^ wide (Fig 1). KSCQZD (M. Aikimbayev’s Kazakh Scientific Center for Quarantine and Zoonotic Diseases) and anti-plague stations carry out the plague surveillance of this territory [14]. The LWC vaccine EV 76 NIIEG is used for plague prophylaxis. Vaccination is administered yearly in springtime among people living in plague-enzootic areas. Public health organizations give recommendations on whom to vaccinate against plague. When plague epizootics are identified, the population of the epizootic areas, as well as veterinarians, farmers, the staff of anti-plague organizations working in the area and tourists visiting the area are vaccinated. The study was conducted between April 15, 2014 and April 15, 2015.

**Fig 2:**
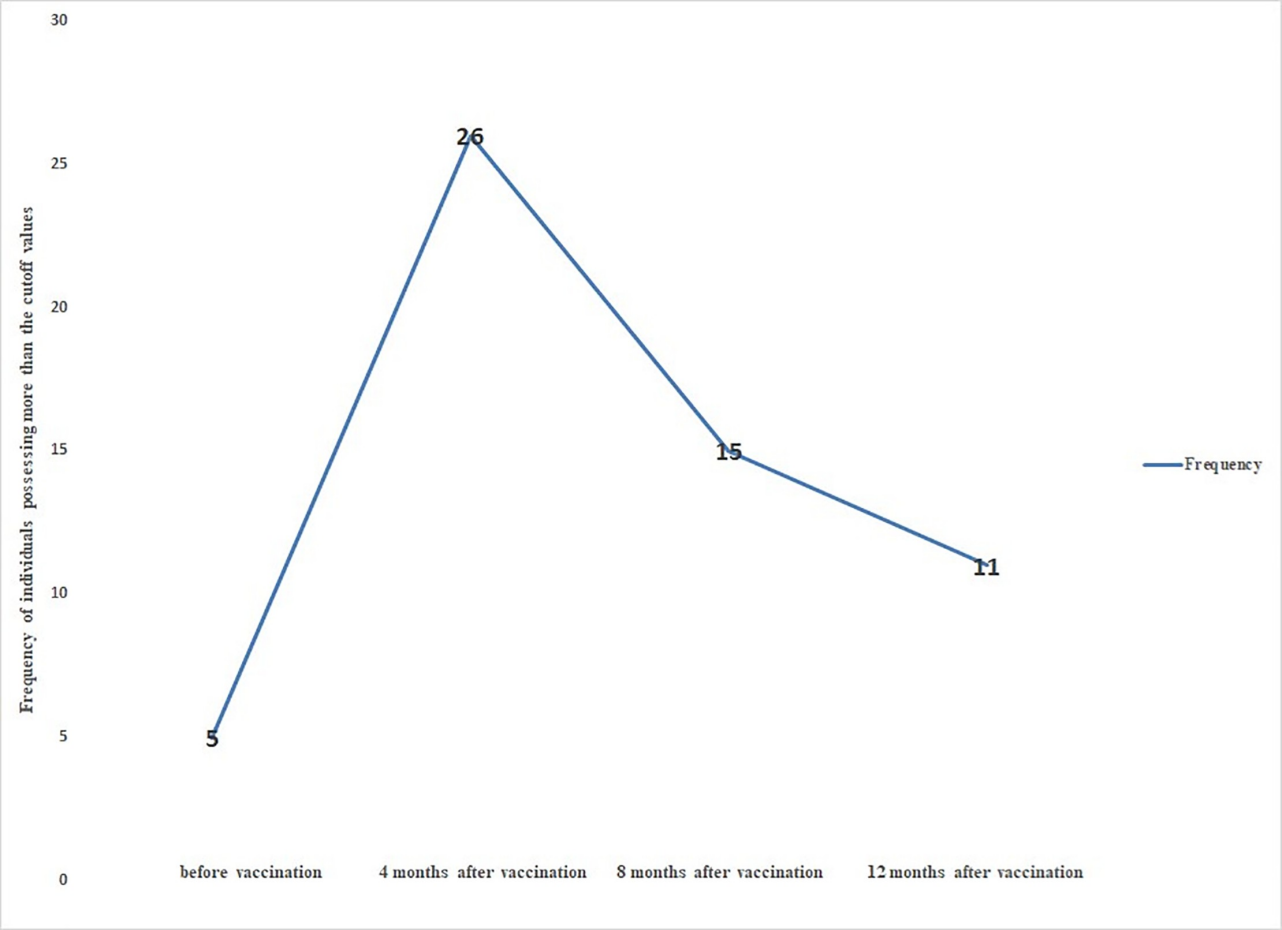
The frequency of individuals possessing more than the cutoff values at different intervals post vaccination.

Among all participants of the study the share of people with a titer of 1:160 and higher was 5% before the vaccination, 26% four months post vaccination, 15% eight months post vaccination, and 11% twelve months post vaccination (Fig 2).

As can be seen from Fig 2, the highest number of patients had titers above 1:160 at four months post-vaccination.

McNemar’s test showed that for those who have not been vaccinated previously the changes in the titer of antibodies were statistically significant at four months after the vaccination (Table 2).

**Table 2:** Statistical data concerning the level of antibodies to F1 *Y*.*pestis* among people have not been vaccinated previously.

Months since vaccination	Odds ratio	95% Confidence Interval LL	95% Confidence Interval UL	*p*-value	Positive/negative results
12 months	4.9	1.1	22.8	0.03	10/164
8 months	6.5	1.5	28.8	0.01	13/154
4 months	14.5	3.5	60.7	0.0003	29/150

For those who have been vaccinated previously the changes in the titer of antibodies were also statistically significant at four months after the vaccination, and then remained statistically significant at 8 and 12 months (Table 3).

**Table 3:** Statistical data concerning the level of antibodies to F1 *Y*.*pesti*s among people have been vaccinated previously.

Months since vaccination	Odds ratio	95% confidence interval LL	95% confidence interval UL	*p*-value	Positive/negative results
12 months	3.8	1.6	9.4	0.003	38/241
8 months	7.2	2.8	18.3	0.0000	52/222
4 months	73.5	10.1	525.2	0.0000	91/184

For both people who have been vaccinated previously and for those who have not, the statistical significance decreases over time.

Table 4 shows the changes in the titer of antibodies over time among all participants of the study.

**Table 4:** Statistical data concerning the level of antibodies to F1 *Y*.*pestis* among all participants of the study.

Months since vaccination	Odds ratio	95% confidence interval LL	95% confidence interval UL	*p*-value	Positive/negative results
12 months	4.1	1.9	4.9	0.0003	48/405
8 months	6.9	3.2	15.4	0.0000	65/376
4 months	33.9	10.8	107.7	0.0000	120/334

Further research was aimed at determining the association if any between positive immune answers and the remaining proposed variables: age, gender, occupation (medical specialist/non-medical specialist), rodent exposure, and level of education. The impact of those variables and its statistical significance 4 months after the vaccination are described in Table 5.

**Table 5:** Multivariate regression analysis of the relationship between proposed variables and serum antibodies to F1 *Y*. *pestis* 4 months after the vaccination.

Variable	Odds ratio	95% CI LL	95% CI UL	*p*-value
Age	0.8555	0.5377	1.3613	0.5102
Education	0.9107	0.5364	1.5463	0.7292
Sex	0.7371	0.4519	1.2024	0.2218
Contact with rodents	0.6492	0.3039	1.3867	0.2646
Occupation (1 group)	1.3741	0.7284	2.5923	0.3264
Occupation (2 group)	0.7679	0.3448	1.7101	0.5179
Vaccinations 5+ years	2.9608	1.4383	6.0951	0.0032
Vaccinations 1–4 years	3.9519	1.7358	8.9975	0.0011

As can be seen from Table 4 that the frequency of vaccination is a statistically significant factor. For those who have been vaccinated 5 or more times previously, the odds ratio is 2.9608, the confidence interval is between 1.4383 and 6.0951, and the p-value is 0.0032. For those who have been vaccinated 1–4 times the odds ratio is 3.9519, the confidence interval is between 1.7358 and 8.9975, and the p-value is 0.0011.

Epidemiologic factors, such as age, level of education, gender, rodent exposure, and occupation had no statistical significance.

In order to determine the association if any between positive immune answers and the proposed variables 8 months after the vaccination we conducted an additional statistical analysis. The results of the analysis are shown in Table 6.

**Table 6:** Multivariate regression analysis of the relationship between proposed variables and serum antibodies to F1 *Y*. *pestis* 8 months after the vaccination.

Parameter	Odds ratio	95% CI LL	95% CI UL	*p*-value
Age	0.9340	0.5195	1.6792	0.8196
Education	0.5478	0.2656	1.1299	0.1033
Sex	0.8481	0.4526	1.5892	0.6072
Contact with rodents	0.4422	0.1431	1.3666	0.1564
Occupation (1group)	0.9373	0.4193	2.0954	0.8746
Occupation (2 group)	1.4098	0.5612	3.5414	0.4649
Vaccinations 5+ years	2.9608	1.4383	6.0951	0.0032
Vaccinations 1–4 years	3.9519	1.7358	8.9975	0.0011

As can be seen from Table 6, the frequency of vaccination is a statistically significant factor among other factors. For those who have been vaccinated 5 or more times the odds ratio is 2.9608, the confidence interval is between 1.4383 and 6.0951, and the p-value is 0.0032. For those who have been vaccinated 1–4 times the odds ratio is 3.9519, the confidence interval is between 1.7358 and 8.9975, and the p-value is 0.0011. Other epidemiologic factors have no statistical significance.

As can be seen from Table 7, the results were different 12 months post vaccination. For those who have been vaccinated 5 or more times the odds ratio is 3.1207, the confidence interval is between 1.3998 and 6.9575, and the p-value is 0.0054. For those who have been vaccinated 1–4 times the odds ratio is 3.2003, the confidence interval is between 1.2368 and 8.2806, and the p-value is 0.0165. Other epidemiologic factors had no statistical significance.

**Table 7:** Multivariate regression analysis of the relationship between proposed variables and serum antibodies to F1 *Y*. *pestis* 12 months after the vaccination.

Parameter	Odds ratio	95% CI LL	95% CI UL	p-value
Age	1.0801	0.5547	2.1034	0.8206
Education	0.6302	0.2831	1.4030	0.2582
Sex	0.7613	0.3688	1.5712	0.4606
Contact with rodents	1.1044	0.3096	3.9403	0.8784
Place of work (1group)	0.6286	0.2247	1.7584	0.3764
Place of work (2 group)	1.6559	0.6063	4.5222	0.3252
Vaccinations 5+ years	3.1207	1.3998	6.9575	0.0054
Vaccinations 1–4 years	3.2003	1.2368	8.2806	0.0165

## Discussion

At this time, there are no alternatives to the existing plague vaccines. Attempts at creating new vaccines are being made around the globe. One such example is the development of a subunit vaccine [6, 9, 15]. In the meantime, the existing plague vaccines will keep being used to protect the population of enzootic areas, the staff of laboratories working with *Y*.*pestis*, and to protect the civilians in case of a biological or terrorist attack using *Y*.*pestis* [10]. The live plague vaccine EV NIIEG saved thousands of lives in the 20^th^ century and is still being used for annual routine vaccination of laboratory staff and the population of enzootic areas in former Soviet states [2,16].

Studying the immune status of people vaccinated against plague remains a relevant field of inquiry in immunology and plague epidemiology because it is valuable both in terms of the theory and practice. In one study, serological methods were used (indirect hemagglutination reaction) to study the serum of 128 people vaccinated with EV vaccine. The serum was taken three months post vaccination. The results showed that antibody titer in serum was between 1:20 and 1:2560. Six months after the vaccination the samples were taken again. This time the antibody titer was 1:20 [16]. Unfortunately, the abstract of the study does not indicate whether the results were obtained through indirect hemagglutination reaction or some other tests. In another study, a group of people was vaccinated three times with KWC. In 7 percent of the vaccinated population, the titer of antibodies did not reach a sufficient level– 128 [17]. Yet another study looked at serum of 30 people who have recovered from plague. Here, the indirect hemagglutination reaction was used. Following results were obtained: all those who have recovered from plague had the antibody titer between 1:80 and 1:81,920, 5–15 days after getting infected. In some of them, the antibody titer remained sufficient for 4–6 years. This study also showed that the antibody titer values among those who have recovered from plague were much higher than among those who have been vaccinated with EV [18].

A different study tested blood serums collected from volunteers vaccinated with the live plague vaccine *EV 76 NIIEG* during the period of 4 to 30 years. The objective of this study was to investigate the period of immunity and the presence of antibodies against specific antigens *F1*, *Pla*, *LcrV*, *YopM*, and *YscF* of *Y*.*pestis*. 14 out of 17 volunteers who participated in the study had at least one antibody against the studied antigens of *Y*.*pestis*. As expected, antibodies to the fraction 1 of *Y*.*pestis* were found in 57% of volunteers. Fewer antibodies were found to the antigens of *LcrV* and *YscF* (26% and 36% respectively). Only 2 volunteers had antibodies to *YopM* (10%) [15]. All the studies aimed at determining the immune status of people vaccinated against plague were either based on single blood sampling or had an insufficient number of subjects. Thus, the field of inquiry remains wide open for research.

Our study demonstrated the statistical difference in antibody levels between the first-time-receivers of the plague vaccine and those who have been vaccinated several times. The first-time-receivers of the plague vaccine had the highest conditionally protective titer of antibodies against the fraction 1 of *Y*.*pestis* at 4 months (OR = 14.5, 95% CI = 3.5–60.7, *p-*value<0.0003) after the vaccination. The level of antibodies fell sharply at 8 months (OR = 6.5, 95% CI = 1.5–28.8, *p-value*<0.01) and further declined at 12 months post vaccination (OR = 4.9, 95% CI = 1.1–28.8, p-value<0.03). The situation was different for those who have been vaccinated in the past. Similar to the first group, the number of serum antibodies to the fraction 1of *Y*.*pestis* peaked at 4 months after the vaccination (OR = 73.5, 95% CI = 10.1–525.2, p-value<0.0000). At 8 months after the vaccination, the number of antibodies went down, but the overall number of people with a conditionally protective titer of antibodies remained statistically significant (OR = 7.2, 95% CI = 2.8–18.3, p-value<0.0000). At 12 months post vaccination, the level of antibodies slightly declined but the outcome still remained statistically significant (OR = 3.8, 95% CI = 1.6–9.4, *p*-value<0.003).

The data from the two groups (first-time-receivers of the vaccine and those who have been vaccinated before) were then combined to obtain overall results. The level of antibodies with a conditionally protective titer (1:160) peaked at 4 months and was statistically significant (OR = 33.9, 95% CI = 10.8–107.7, *p-*value<0.0000). Despite the decrease at 8 months after the vaccination, the serum antibodies remained statistically significant (OR = 6.9, 95% CI = 3.2–15.4, *p-*value<0.0000). At 12 months the level of antibodies declined slightly but remained statistically significant (OR = 4.1, 95% CI = 1.9–4.9, p-value<0.0003). People vaccinated against plague several times kept a level of antibody that represents a conditionally protective titer against the fraction 1 of *Y*.*pestis* longer compared to the first-time-receivers of the vaccine. To verify the obtained data we conducted a multivariable analysis, which showed that the frequency of vaccination was a statistically significant factor. The analysis also showed that age, gender, occupation, rodent exposure, and education did not significantly affect the level of antibodies against the fraction 1 of *Y*.*pestis*.

Following results were obtained in our study: in all vaccinated people the highest level of antibody that represents a conditionally protective titer against F1 *Y*.*pestis* was observed 4 months after the vaccination with EV 76 NIIEG; among those who have been vaccinated prior to the study the level of antibodies remained conditionally protective for a longer period of time; the number of vaccines received affected the level of antibodies against F1 *Y*.*pestis*.

Based on the results of this study, we recommend initiating vaccination campaigns for the medical and veterinary staff, as well as the general population four months prior to the springtime epizootics of plague among wild rodents.

## Limitations

Due to financial constraints, we used indirect hemagglutination reaction for detecting antibodies against capsular antigen *Y*.*pestis*. In the future, we plan to conduct a similar study using immunoassay analysis. Indirect hemagglutination reaction allowed us to detect antibodies only against F1 *Y*. *pestis*, while immunoassay analysis could help detect other *Y*. *pestis* antigens, *Pla*, *LcrV*, *YopM*, and *YscF*.

Initially, this study was going to be conducted in three regions of Kazakhstan. Due to time constraints, it was conducted only in one region.

## Supporting information

S1 Fig During conducting of the field study “Human response to live plague vaccine EV, Almaty region, Kazakhstan, 2014–2015. (TIF)

S2 Fig Fragment of results of Indirect hemagglutination reaction with antigenic erythrocyte assay (micromethod) for identifying antibodies to F1 *Y*.*pestis*. (TIF)

S1 Table Fragment of database of study “Human response to live plague vaccine EV, Almaty region, Kazakhstan, 2014–2015. (PDF)

## References

[1] Adamovicz JJ, Andrews GP. Plague Vaccines Retrospective Analysis and Future Developments. Biological Weapons Defense Part of the series Infectious Disease 2005; 121–151. Available: https://link.springer.com/chapter/10.1385/1-59259-764-5:121

[2] Meyer KF. Effectiveness of Live or Killed Plague Vaccines in Man. Bulletin World Health Organization 1970; 42: 653–666. Available: https://www.ncbi.nlm.nih.gov/pmc/articles/PMC2427500/PMC2427500 4988692

[3] Perry RD, Fetherston JD. *Yersinia pestis*–etiologic agent of plague. *Clin Microbiol Rev*. 1997; 10:35–66. Available: http://cmr.asm.org/content/10/1/35.short 8993858 10.1128/cmr.10.1.35 PMC172914

[4] Sabhnani L, Rao DN. Identification of immunodominant epitope of F1 antigen of *Yersinia pestis*. FEMS immunology and Medical Microbiology 2000; 27 (2): 155–162. Available: https://academic.oup.com/femspd/article/27/2/155/507498 10.1111/j.1574-695X.2000.tb01426.x 10640611

[5] Feodorova VA, Motin VL. Plague vaccines: current developments and future perspectives. Emerging Microbes and Infections 2012; 1 (11) e36: 10.1038/emi.2012.34 Available: https://www.nature.com/articles/emi201234 26038406 PMC3630923

[6] Dentovskaya SV, Kopylov Pkh, Ivanov SA, Ageev SA, Anisimov AP. Molecular Bases of Vaccine Prevention of Plague. Molecular Genetic, Microbiology and Virology 2013; 28 (3):87–98. Available: https://link.springer.com/article/10.3103/S089141681303004X

[7] Feodorova VA, Khizhnyakova MA, Lyapita AM, et al Selectivity in IgG subclass Response to Live Plague Vaccine in Humans. *Procedia in Vaccinology* 2014; 8: 34–36. Available: https://www.sciencedirect.com/science/article/pii/S1877282X14000071

[8] Feodorova VA, Sayapina LV, Corbel MJ, Motin VL. Russian vaccines against especially dangerous bacterial pathogens. Emerging Microbes and Infections 2014b; 3, e86: 10.1038/emi.2014.82 Available: https://www.nature.com/articles/emi201482 26038506 PMC4317636

[9] Nazarova EL, Dyatlov IA, Pozdeev NM, Demyanova VT, Paramonov IV, Rylov AV, et al Genetic markers of immune response to *Yersinia pestis F1* and V Antigens–loaded microspheres vaccine against plague. Russian Biomedical Research 2017; 2(1):19–28. Available:https://www.researchgate.net/profile/Andrey_Anisimov/publication/318283908_Genetic_markers_of_immune_response_to_Yersinia_pestis_F1_and_V_antigens.

[10] Titball RW & Williamson ED *Yersinia pestis* (plague) vaccines. Expert Opinion on Biological Therapy 2004; 4(6): 965–973. Available: 10.1517/14712598.4.6.965 15174978

[11] Blisnick T, Ave P, Huerre M, Carniel E, Demeure CE. Oral Vaccination against bubonic plague using a live avirulent *Yersinia pseudotuberculosis* strain. Infection and Immunity 2008; 76(8): 3808–3816. Available: http://iai.asm.org/content/76/8/3808.short 10.1128/IAI.00034-08 18505804 PMC2493205

[12] Plague Vaccine. Morbidity and Mortality Weekly Report (MMWR) 1982; 31(22):301–304 Available: https://www.cdc.gov/mmwr/preview/mmwrhtml/00041848.htm 6810087

[13] Epi InfoTM program. Available: (https://www.cdc.gov/epiinfo/index.html.

[14] Aikimbayev Alim M., Bekenov Zhumabek Y., Meka-Mechenko Tatyana V., Gulnara A. Temiraliyeva The epidemiological surveillance of Highly Pathogenic Diseases in Kazakhstan. Emerging and Endemic Pathogens 2010; 15–20: https://www.springer.com/la/book/9789048196364

[15] Feodorova VA, Layapina AM, Ulianova O.V., Layapina EP, Sayapina LV, Layapin MN, et al Serological Markers for Long-Term Immunity in Humans Vaccinated with Live *Yersinia pestis* EV NIIEG. *Procedia in Vaccinology* 2012b; 6: 10–13 Available: https://www.sciencedirect.com/science/article/pii/S1877282X12000057

[16] Hui-fan Y. Observation on the serological responses of persons and guinea pigs after vaccination with EV strain of *Yersinia pestis* and the guinea pig protection test. *Endemic Disease Bulletin* 1986–03: Available: http://en.cnki.com.cn/Article_en/CJFDTOTAL-DFBT198603008.HTM

[17] Wang X, Zhang X., Zhou D., Yang R. Live-attenuated *Yersinia pestis* vaccines. Expert Rev Vaccines 2013; 2 (6): 677–686. Available: https://www.tandfonline.com/doi/abs/10.1586/erv.13.42 10.1586/erv.13.42 23750796

[18] Zhu Jin-xin Chen Yao-hui. Analysis of indirect hemagglutination test on thirty patients suffering from plague. Endemic Disease Bulletin 1986–01: Available: http://en.cnki.com.cn/Article_en/CJFDTotal-DFBT198601007.htm

