# Supplementary figures and images for: Human response to live plague vaccine EV, Almaty region, Kazakhstan, 2014-2015
Source: PLoS One. 2019 Jun 14;14(6):e0218366. doi: 10.1371/journal.pone.0218366 (PMC6568420; doi:10.1371/journal.pone.0218366)

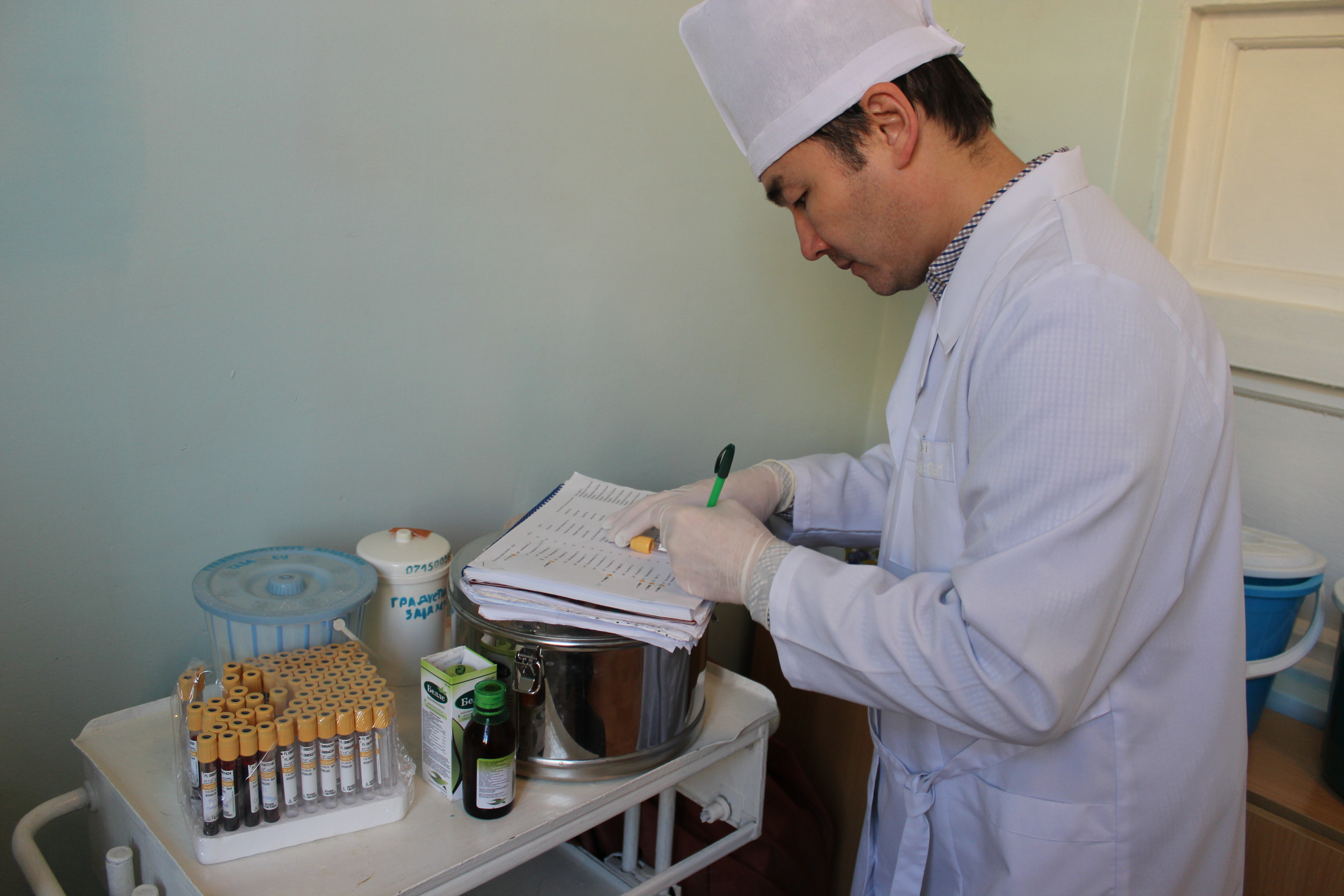

Supplement: S1 Fig — (TIF) [file pone.0218366.s001.tif]

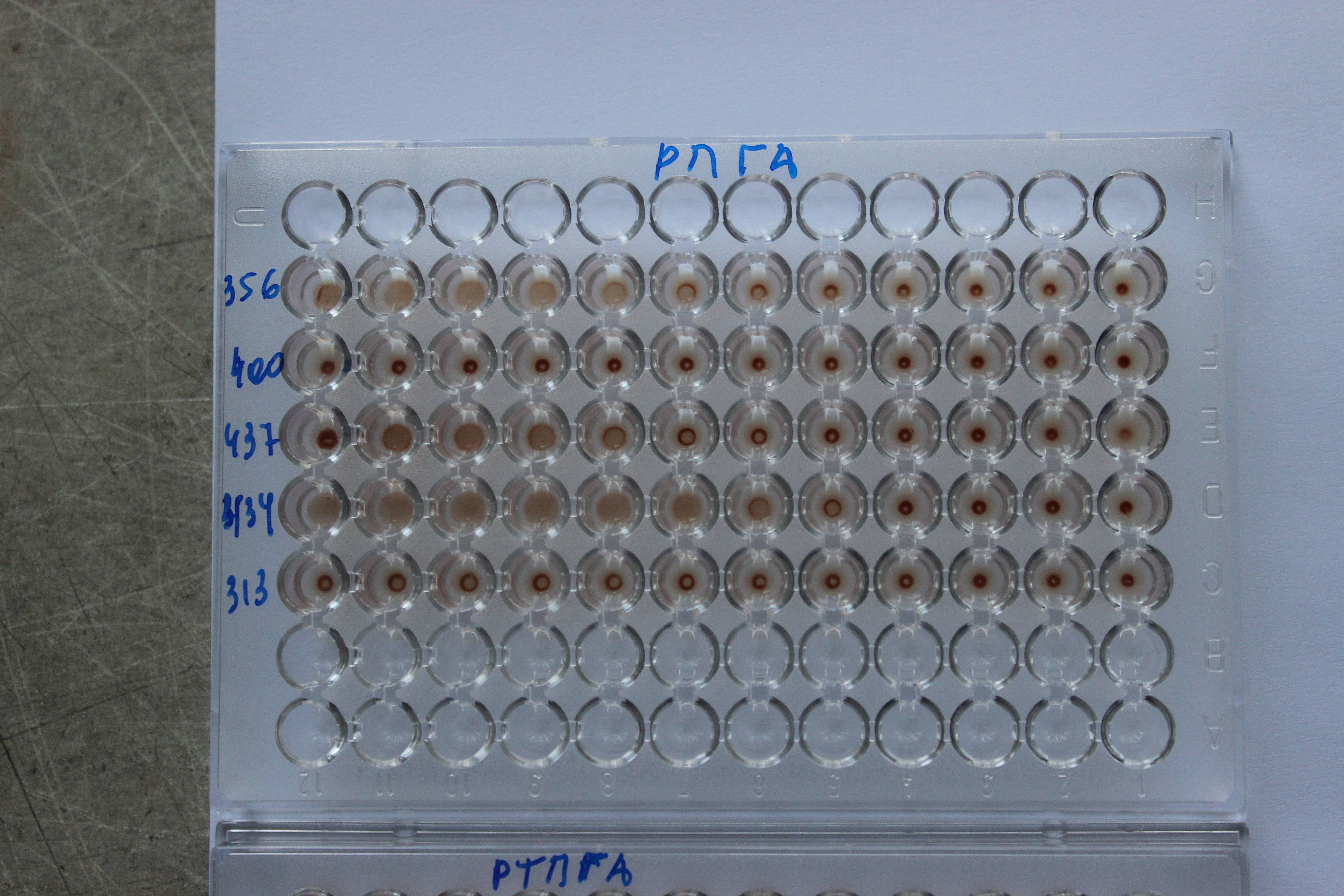

Supplement: S2 Fig — (TIF) [file pone.0218366.s002.tif]
