# Supplementary material for: Human response to live plague vaccine EV, Almaty region, Kazakhstan, 2014-2015
Source: PLoS One. 2019 Jun 14;14(6):e0218366. doi: 10.1371/journal.pone.0218366 (PMC6568420; doi:10.1371/journal.pone.0218366)
Supplement: S1 Table — (PDF) [file pone.0218366.s003.pdf]

S1 Table. Fragment of database of study “Human response to live plague vaccine EV, Almaty region, Kazakhstan, 2014-2015

	A	B	C	D	E	F	G	H	I	J	K	L	M	N	O	P	
1	uid	restfirst	restsecond	restthird	restfourth	agegrp	educgrp	gender	workplace	vaccprevfreq	rodentcontact	vacc2014	vacc13121110	restfirst14	restsecond14	restthird14	res
2	1	0	0	0	0	1	1	0	1	2	0		1				
3	2	0	0	0	0	1	0	0	1	2	0		1				
4	3	0	0	0	0	1	0	1	1	2	1		1				
5	4	0				0	0	1	1	0	0	1		0			
6	5	0	1	0	0	1	0	1	1	2	0		1				
7	6	0				1	0	1	1	0	0	1		0			
8	7	0	0	0	0	1	0	1	1	2	1		1				
9	8	0	0	0	0	0	0	1	1	2	1		1				
10	9	0	0	0	0	0	0	1	1	2	0		1				
11	10	0	0	0	0	1	0	1	1	2	0		1				
12	11	0	0	0	0	0	0	1	1	0	0	1		0	0	0	
13	12	0	0	0	0	1	0	1	1	2	1		1				
14	13	0	0	0	0	0	0	1	1	2	0		1				
15	14	0	1	0	0	0	0	1	1	2	1		1				
16	15	0	0	0	0	0	1	0	1	2	1		1				
17	16	0	0	0	0	0	1	0	1	2	1		1				
18	17	0	0	0	0	0	1	0	1	1	1		1				
19	18	0	0	0	0	0	0	0	1	2	1		1				
20	19	0	0	0	0	0	0	0	1	2	1		1				
21	20	0	0	0	0	1	0	0	1	2	1		1				
22	21	0	0	1	1	1	0	1	1	2	1		1				
23	22	0	0	0	0	1	1	1	1	2	1		1				
24	23	0	0	0	0	0	1	0	1	1	1		1				
25	24	1	1	1	1	1	0	1	1	2	0		1				
26	25	0	0	0	0	1	0	1	1	2	1		1				
27	26	0	1	0	0	0	1	1	1	2	1		1				
28	27	0	0	0	0	0	1	1	1	1	1		1				
29	28	0	0	0	0	0	0	1	1	1	1		1				
30	29	0	1	0	0	0	1	1	1	1	1		1				
31	30	0	0	0	0	1	0	0	1	2	1		1				
32	31	0	0	0	0	1	0	0	1	2	1		1				
33	32	0	0	0	0	0	0	0	1	2	1		1				
34	33	0	1	1	1	1	0	0	1	2	0		1				
35	34	0	0	0	0	1	1	0	1	2	0		1				
36	35	0	1	1	1	1	0	0	1	2	0		1				
37	36	0	1	0	0	1	0	0	1	2	1		1				
38	37	0	1	1	1	0	0	0	1	2	0		1				
39	38	0	0	0	0	0	0	0	1	1	1		1				
40	39	0	0	0	0	1	0	0	1	2	1		1				
41	40	0	0	1	0	0	1	1	1	2	1		1				
42	41	0	0	0	0	0	1	1	1	1	0		1				
43	42	0	0	0	0	0	1	1	1	1	0		1				
44	43	0	1	0	0	0	1	0	1	2	0		1				
45	44	0	0	0	0	0	0	0	1	0	0	1		0	0	0	
46	45	0	1	0	0	1	1	0	1	1	0		1				
47	46	0	0	0	0	0	0	0	1	2	0		1				
48	47	0	0	0	0	1	0	0	1	1			1				
49	48	0	1	0	0	0	0	0	1	2	0		1				
50	49	0	1	1	0	1	0	0	1	2	0		1				
51	50	0	1	1	0	1	0	1	1	2	0		1				
52	51	0	0	0	0	1	0	0	1	1	0		1				
53	52	0	1	0	0	1	0	0	1	1	0		1				
54	53	0	0	0	0	1	1	0	1	2	1		1				
55	54	0	1	0	0	1	1	1	1	2	1		1				
56	55	0	0	0	0	1	1	1	1	2	1		1				
57	56	0	0	0	0	1	0	1	1	1	1		1				
58	57	0	1	0	0	0	0	1	1	2	1		1				
59	58	0	0	0	0	0	0	1	1	0	0	1		0	0	0	
60	59	0	1	1	0	1	0	1	1	2	0		1				
61	60	0	0	0	0	0	1	1	1	1	0		1				
62	61	0	0	0	0	1	0	1	1	2	1		1				
63	62	0	0	0	0	0	0	1	1	1	0		1				
64	63	0	1	0	0	0	0	1	1	0	0	1		0	1	0	
65	64	0	0	0	0	0	1	1	1	1	0		1				
66	65	0	0	0	0	0	1	1	1	0	0	1		0	0	0	
67	66	0	0	0	0	0	1	1	1	1	0		1				
68	67	0	1	0	0	0	0	1	1	1	0		1				
69	68	0	0	1	1	0	1	1	1	1	0		1				
70	69	0	0	0	0	1	0	1	1	2	0		1				
71	70	0	0	0	0	1	0	1	1	2	1		1				
72	71	0	1	0	0	0	0	1	1	1	0		1				
73	72	0	0	0	0	1	0	1	1	2	0		1				
74	73	0	1	0	0	0	0	1	1	1	0		1				
75	74	0	0	0	0	1	0	1	1	2	0		1				
76	75	0	1	0	0	1	0	1	1	2	0		1				
77	76	0	0	0	0	0	0	1	1	2	0		1				
78	77	0	1	0	0	0	0	1	1	1	0		1				
79	78	0	0	0	0	0	1	1	1	1	0		1				
80	79	0	0	0	0	0	1	1	1	0	0	1		0	0	0	
81	80	0	0	0	0	1	0	1	1	2	0		1				
82	81	0	1	0	0	0	1	1	1	1	0		1				
83	82	0	1	0	0	0	0	1	1	2	0		1				
84	83	0	1	1	0	0	0	1	1	1	0		1				
85	84	0	0	0	0	0	0	1	1	1	0		1				
86	85	0	0			1	0	1	1	1	0		1				
87	86	0	1	1	0	1	0	1	1	2	0		1				
88	87	0	0	0	0	1	0	1	1	2	0		1				
89	88	0	0	0	0	0	0	0	1	1	0		1				
90	89	0	0	0	0	1	0	0	1	2	0		1				
91	90	0	0	0	0	0	1	1	1	2	0		1				
92	91	0	0	0	0	0	0	0	1	2	1		1				
93	92	0	0	0	0	0	0	1	1	1	0		1				
94	93	0	0	0	0	0	0	1	1	2	0		1				
95	94	0	0	0	0	1	0	0	1	2	0		1				
96	95	0	0	0	0	0	0	1	1	2	1		1				
97	96	0	1	1	0	0	0	1	1	2	0		1				
98	97	1	1	1	1	0	0	0	1	2	1		1				
99	98	0	0	0	0	0	0	1	1	2	0		1				
100	99	0	0	0	0	1	0	1	1	2	0		1				
101	100	0	0	0	0	1	0	1	1	2	0		1				
102	101	0	1	1	0	1	0	1	1	2	0		1				
103	102	0	0	0	0	1	1	1	1	2	1		1				
104	103	0	0	0	0	1	1	1	1	2	0		1				
105	104	0	0	0	0	1	0	0	1	1	0		1				
106	105	0	1	0	0	1	1	1	1	2	1		1				
107	106	0	1	0	0	1	1	1	1	2	1		1				
108	107	0	1	0	0	0	0	1	1	1	0		1				
109	108	0	1	0	0	0	1	0	1	2	0		1				
110	109	0	1	1	1	1	0	0	1	2	1		1				

111	110	0	0	0	0	0	1	0	1	2	1		1			
112	111	0	1	0	0	1	1	0	1	2	1		1			
113	112	0	1	1	1	1	1	0	1	2	1		1			
114	113	0	0			1	1	1	1	2	1		1			
115	114	0	0	0	0	1	0	0	1	2	1		1			
116	115	0	0			1	0	0	1	2	1		1			
117	116	0	0			1	0	0	1	2	1		1			
118	117	0	0			0	0	0	1	0	0	1		0	0	
119	118	0	0			1	0	0	1	2	0		1			
120	119	0	0			1	0	1	1	1	1		1			
121	120	0	0			1	0	1	1	0	0	1		0	0	
122	121	0	0			1	1	1	1	2	1		1			
123	122	0	0			1	0	1	1	2	1		1			
124	123	0	0			1	1	1	1	2	0		1			
125	124	0	0			1	0	1	1	2	1		1			
126	125	0	0			1	0	1	1	0	0	1		0	0	
127	126	0				1	1	1	1	2	0		1			
128	127	0				0	0	1	1	1	1		1			
129	128	0	0			1	0	1	1	1	0		1			
130	129	0	1	1	1	1	0	1	1	2	0		1			
131	130	0	0	0	0	0	1	1	1	1	0		1			
132	131	0	1	0	0	0	1	1	1	2	1		1			
133	132	0	1	0	0	0	1	0	1	0	1	1		0	1	0
134	133	0	1	0	1	0	0	0	1	2	1		1			
135	134	0	1	0	0	0	1	0	1	2	0		1			
136	135	0	1	0	0	0	0	1	1	1	1		1			
137	136	0	0	0	0	0	1	1	1	2	1		1			
138	137	0	1	0	0	1	1	1	1	2	1		1			
139	138	0	0	0	0	1	0	0	0	1	0		1			
140	139	0	0	0	0	1	0	0	0	2	0		1			
141	140	0	0	0	0	1	0	0	0	2	0		1			
142	141	0	0	0	0	1	0	0	0	2	0		1			
143	142	0	0	0	0	1	0	0	0	2	0		1			
144	143	0	0	0	0	1	0	0	0	2	0		1			
145	144	0	0	0	0	1	0	0	0	2	0		1			
146	145	0	0	0	0	0	0	0	0	0	0	1		0	0	0
147	146	0	0	0	0	0	0	0	0	1	0		1			
148	147	0	0	0	0	0	0	0	0	1	0		1			
149	148	0	0	1	1	1	0	0	0	1	0		1			
150	149	0	0	0	0	1	0	0	0	2	0		1			
151	150	0	0	0	0	1	0	0	0	2	0		1			
152	151	0	0	0	0	1	0	0	0	2	0		1			
153	152	0	1	0	0	1	0	0	0	2	0		1			
154	153	0	1	1	1	0	0	0	0	2	0		1			
155	154	0	1	1	1	1	0	0	0	1	0		1			
156	155	0	0	0	0	1	1	1	0	1	0		1			
157	156	0	0	0	0	1	0	0	0	2	0		1			
158	157	0	1	0	0	0	0	0	0	1	0		1			
159	158	0	0	0	0	0	0	0	0	2	0		1			
160	159	0	1	1	1	1	0	0	0	1	0		1			
161	160	0	1	1	1	1	0	0	0	2	0		1			
162	161	0	0	1	0	0	0	0	0	2	0		1			
163	162	0	0	0	0	1	0	0	0	2	0		1			
164	163	0	0	0	0	1	1	1	2	1	0		1			
165	164	0	1	0	0	0	1	1	2	0	0	1		0	1	0
166	165	0	0	0	0	0	1	1	2	0	0	1		0	0	0
167	166	1	1	1	1	0	1	1	2	1	0		1			
168	167	0	1	0	0	1	0	1	2	1	0		1			
169	168	0	0	0	0	1	1	1	2	1	0		1			
170	169	0	0	0	0	0	1	1	2	1	0		1			
171	170	0	0	0	0	1	0	1	2	1	0		1			
172	171	0	0	1	0	1	1	1	2	1	0		1			
173	172	0				0	1	1	2	1	0		1			
174	173	0	0	0	0	0	1	1	2	1	0		1			
175	174	0	1	0	0	1	0	1	2	1	0		1			
176	175	0	0	0	0	1	1	1	2	0	0	1		0	0	0
177	176	0	1	0	0	1	1	0	2	0	0	1		0	1	0
178	177	0	0	0	0	1	0	1	2	1	0		1			
179	178	0	1	0	0	0	1	1	2	0	0	1		0	1	0
180	179	0	0	0	0	0	1	1	2	1	0		1			
181	180	0	0	0	0	0	1	1	2	0	0	1		0	0	0
182	181	0				0	0	1	2	1	0		1			
183	182	0	0	1	1	0	1	1	2	0	0	1		0	0	1
184	183	0	0	0	0	0	1	1	2	0	0	1		0	0	0
185	184	0	0	0	0	0	1	1	2	0	0	1		0	0	0
186	185	1	1	0	0	1	1	1	2	1	0		1			
187	186	0	0	0	0	1	1	1	2	1	0		1			
188	187	0	0	1	1	0	1	1	2	1	0		1			
189	188	1	1	1	1	0	1	1	2	1	0		1			
190	189	0	0	0	0	1	0	0	2	0	0	1		0	0	0
191	190	0	0	0	0	1	0	1	2	1	0		1			
192	191	0	0	1	0	0	0	1	2	0	0	1		0	0	1
193	192	0	0	0	0	0	1	1	2	1	0		1			
194	193	1	1	1	1	0	1	1	2	1	0		1			
195	194	0	0	0	0	0	0	1	2	0	0	1		0	0	0
196	195	0	0	0	0	1	0	1	2	1	0		1			
197	196	0	0	0	0	0	0	1	2	1	0		1			
198	197	0	0	0	0	0	0	1	2	0	0	1		0	0	0
199	198	0				0	0	1	2	0	0	1		0		
200	199	0	0	0	0	1	1	1	2	1	0		1			
201	200	0	0	0	0	0	0	1	2	0	0	1		0	0	0
202	201	0	0	0	0	1	0	1	2	0	0	1		0	0	0
203	202	1	0	0	1	1	1	1	2	1	0		1			
204	203	0		0	0	0	0	1	2	1	0		1			
205	204	0	0	0	0	0	0	1	2	1	0		1			
206	205	0	0	0	0	0	0	1	2	0	0	1		0	0	0
207	206	0	0	0	0	1	0	1	2	0	0	1		0	0	0
208	207	0	0	0	0	0	1	1	2	1	0		1			
209	208	0	0	0	0	0	0	1	2	1	0		1			
210	209	0	0	0	0	1	0	1	2	1	0		1			
211	210	0		0	0	1	0	1	2	1	0		1			
212	211	0	0	0	0	0	1	1	2	1	0		1			
213	212	0	0	0	0	0	1	1	2	0	0	1		0	0	0
214	213	0	0	0	0	0	1	1	2	0	0	1		0	0	0
215	214	0		0	0	1	0	1	2	1	0		1			
216	215	0	0	0	0	0	1	1	2	0	0	1		0	0	0
217	216	0				1	0	1	2	1	0		1			
218	217	1	1	1	1	1	0	1	2	1	0		1			
219	218	0	0	0	0	1	0	1	2	0	0	1		0	0	0
220	219	0	0			1	0	1	2	0	0	1		0	0	
221	220	0	1	1	1	0	1	1	2	1	0		1			
222	221	0	0		0	1	0	1	2	0	0	1		0	0	
223	222	0	0		0	0	1	1	2	1	0		1			
224	223	0	0	0	0	1	0	1	2	1	0		1			
225	224	0	0	0	0	0	0	1	2	1	0		1			
226	225	0				1	0	1	2	1	0		1			
227	226	0	0	0	0	0	1	1	2	0	0	1		0	0	0
228	227	0				1	1	1	2	2	0		1			

228	0			0	0	1	1	2	0	0	1		0		
229	0				0	1	1	2	1	0		1			
230	0	0	0	0	0	0	1	2	1	0		1			
231	0	1	0	0	1	0	0	2	0	0	1		0	1	0
232	0	0	0	0	0	1	0	2	0	0	1		0	0	0
233	0			0	1	1	1	2	1	0		1			
234	0	0	0	0	1	0	1	2	1	0		1			
235	0	0	0	0	1	1	1	2	2	0		1			
236	0	0	0	0	1	0	0	0	2	0		1			
237	0	0	0	0	1	0	0	0	2	0		1			
238	0		0	0	1	1	0	0	2	0		1			
239	0	0	1	0	1	0	0	0	1	0		1			
240	0	1	1	0	1	0	0	0	2	0		1			
241	0	0	0	0	0	1	0	0	1	0		1			
242	0	0	0	0	1	0	0	0	2	0		1			
243	0				0	0	0	0	0	0	1		0		
244	0				0	1	0	0	1	0		1			
245	0	0	0	0	0	1	0	0	0	0	1		0	0	0
246	0	1	0	0	1	0	0	0	1	0		1			
247	0	1	1	0	1	0	0	0	2	0		1			
248	0		0	0	0	1	0	0	2	0		1			
249	1				1	1	0	0	2	0		1			
250	0			0	1	0	1	0	1	0		1			
251	0			0	1	0	0	0	1	0		1			
252	0	1	0	1	1	0	1	0	1	0		1			
253	0	0	0	0	1	0	0	0	1	0		1			
254	0	1	0	0	1	0	0	0	1	0		1			
255	0	1	0	0	1	0	0	0	1	0		1			
256	1		1	0	0	0	0	0	1	0		1			
257	0	1	0	0	1	0	0	0	1	0		1			
258	0	1	0	0	1	0	0	0	1	0		1			
259	0	0	0	0	1	0	1	0	0	0	1		0	0	0
260	0	0	0	0	1	0	0	0	1	0		1			
261	0	1	0	0	0	0	0	0	1	0		1			
262	0	0	0	0	1	0	0	0	1	0		1			
263	0	1			0	0	0	0	0	0	1		0	1	
264	0	0	0	0	1	0	1	0	1	0		1			
265	0	0	0	0	0	0	0	0	1	0		1			
266	1	1	1	0	1	0	0	0	1	0		1			
267	0				1	0	0	0	1	0		1			
268	0	0	0	0	1	0	1	0	1	0		1			
269	0	0	0	0	1	1	0	0	1	0		1			
270	1	1	1	1	1	0	0	0	1	0		1			
271	0	1	1	1	1	0	0	0	1	0		1			
272	0	0	0	0	1	0	1	0	1	0		1			
273	0	0	0	0	1	0	0	0	1	0		1			
274	0	0	0	0	1	0	0	0	1	0		1			
275	1	1	1	1	0	0	0	0	1	0		1			
276	1	1	1	0	1	0	0	0	1	0		1			
277	0	1	1	0	1	0	0	0	1	0		1			
278	0	1	0	0	1	0	0	0	1	0		1			
279	0	1	0	0	1	1	0	0	1	0		1			
280	0	0	0	0	1	0	1	0	1	0		1			
281	0	0	0	0	1	0	0	0	1	0		1			
282	1	1	1	0	0	0	1	0	1	0		1			
283	0	1	1	0	0	0	1	0	1	0		1			
284	0	1	0	0	0	0	1	0	1	0		1			
285	1	1	1	1	1	0	0	0	1	0		1			
286	0	0	0	0	0	0	1	0	1	0		1			
287	0		0	0	0	1	0	0	1	0		1			
288	0		0	0	1	0	0	0	1	0		1			
289	0	0		0	0	1	0	0	0	0	1		0	0	
290	0	0	0	0	1	0	1	0	1	0		1			
291	0	1	0	0	0	1	1	0	1	0		1			
292	0		0	0	1	1	0	0	1	0		1			
293	0	0	0	0	0	0	0	0	1	0		1			
294	0	0	0	0	1	0	0	0	1	0		1			
295	1		0	1	0	0	0	0	1	0		1			
296	0	1	0	1	1	0	1	0	1	0		1			
297	0	0	0	0	0	1	1	0	0	0	1		0	0	0
298	0		0	0	1	0	0	0	1	0		1			
299	0	1	0	0	1	0	0	0	0	0	1		0	1	0
300	0	0	0	0	1	0	1	2	0	0	1		0	0	0
301	0	0	0	0	0	0	0	0	0	0	1		0	0	0
302	0	0	0	0	1	0	0	0	1	0		1			
303	0	0	0	0	1	0	1	0	0	0	1		0	0	0
304	0	0	0	0	1	0	0	0	0	0	1		0	0	0
305	0	0	0	0	1	0	0	0	0	0	1		0	0	0
306	0	0	0	0	1	1	0	0	0	0	1		0	0	0
307	0	0	0	0	0	0	0	0	0	0	1		0	0	0
308	0	0	0	0	0	0	0	0	0	0	1		0	0	0
309	0	0	0	0	1	0	0	0	0	0	1		0	0	0
310	0	0	0	0	0	0	1	0	0	0	1		0	0	0
311	0	0	0	0	1	0	0	0	0	0	1		0	0	0
312	0	1	0	0	1	0	0	0	0	0	1		0	1	0
313	0	0	0	0	1	0	0	0	0	0	1		0	0	0
314	0	0	0	0	1	0	1	0	0	0	1		0	0	0
315	0	0	0	0	0	0	0	0	0	0	1		0	0	0
316	0	0	0	0	1	1	0	0	0	0	1		0	0	0
317	0	0	0	0	0	1	0	0	0	0	1		0	0	0
318	0	0	0	0	0	0	1	0	0	0	1		0	0	0
319	0	0	0	0	1	0	0	0	0	0	1		0	0	0
320	0		0	0	1	0	0	0	0	0	1		0		0
321	0	0	0	0	0	1	0	0	0	0	1		0	0	0
322	0	0	0	0	1	0	0	0	0	0	1		0	0	0
323	0	0	0	0	1	0	0	0	1	0		1			
324	0	0	0	0	1	0	0	0	1	0		1			
325	0	0	0	0	0	0	0	0	0	0	1		0	0	0
326	0	1	0	0	1	0	1	0	0	0	1		0	1	0
327	0		0	0	0	0	0	2	0	0	1		0		0
328	0	1	0	0	0	0	0	0	0	0	1		0	1	0
329	0	0	0	0	0	0	0	0	0	0	1		0	0	0
330	0	0	0	0	1	0	0	0	0	0	1		0	0	0
331	0	0	0	0	1	0	1	0	0	0	1		0	0	0
332	0	0	0	0	0	0	0	0	0	0	1		0	0	0
333	0	1	1	1	0	0	0	0	0	0	1		0	1	1
334	0	0	0	0	1	1	1	0	0	0	1		0	0	0
335	0	1	0	0	0	1	1	0	1	0		1			
336	0	0	0	0	1	0	1	0	0	0	1		0	0	0
337	0	1	0	0	1	0	0	0	0	0	1		0	1	0
338	0	1	0	0	1	0	0	0	0	0	1		0	1	0
339	0	1		0	0	1	0	0	0	0	1		0	1	
340	0	0			1	0	0	0	0	0	1		0	0	
341	0	0	0	0	1	0	0	0	0	0	1		0	0	0
342	0	0	0	1	1	0	1	0	1	0		1			
343	0	0	0	0	1	0	1	0	0	0	1		0	0	0
344	0	0	0	0	1	0	1	0	0	0	1		0	0	0
345	0	0	0	0	0	1	0	0	0	0	1		0	0	0
346	0	0	0	0	1	0	0	0	0	0	1		0	0	0

348	347	0	0	0	0	1	0	0	0	0	0	1		0	0	0
349	348	0	0	0	0	0	0	0	0	0	0	1		0	0	0
350	349	0	1			0	0	0	0	0	0	1		0	1	
351	350	0	0	0	0	0	0	0	0	0	0	1		0	0	0
352	351	0	0	0	0	1	0	1	0	0	0	1		0	0	0
353	352	0	0	0	0	0	1	0	0	0	0	1		0	0	0
354	353	0	0	0	0	0	1	1	0	0	0	1		0	0	0
355	354	0	0	0	0	1	0	0	0	0	0	1		0	0	0
356	355	0	0	0	0	1	0	0	0	0	0	1		0	0	0
357	356	0	0	1	1	1	0	1	2	0	0	1		0	0	1
358	357	0	0	0	0	1	0	0	0	0	0	1		0	0	0
359	358	0	0	0	0	1	0	0	0	0	0	1		0	0	0
360	359	0	0	0	0	1	0	1	0	0	0	1		0	0	0
361	360	0	1	0	0	0	1	0	0	0	0	1		0	1	0
362	361	0	0	0	0	1	0	0	0	0	0	1		0	0	0
363	362	0	0	0	0	0	0	0	0	1	0		1			
364	363	0	0	0	0	0	0	0	0	0	0	1		0	0	0
365	364	0	0	0	0	0	1	1	0	0	0	1		0	0	0
366	365	0	0	0	0	0	0	1	2	0	0	1		0	0	0
367	366	0	0	0	0	0	0	1	0	0	0	1		0	0	0
368	367	0	0	0	0	1	0	0	0	1	0		1			
369	368	0	0	0	0	0	0	0	0	0	0	1		0	0	0
370	369	0	0	0	0	1	0	0	0	0	0	1		0	0	0
371	370	0	1	0	0	1	0	0	0	0	0	1		0	1	0
372	371	0	0	0	0	1	0	0	0	0	0	1		0	0	0
373	372	1	0	0	0	1	0	0	0	0	0	1		1	0	0
374	373	0	0	0	0	0	1	1	0	0	0	1		0	0	0
375	374	0	0	0	0	0	0	0	0	0	0	1		0	0	0
376	375	0	0	0	0	1	1	0	0	0	0	1		0	0	0
377	376	0	0	0	0	0	0	0	0	0	0	1		0	0	0
378	377	0	1	0	0	1	1	0	0	0	0	1		0	1	0
379	378	0				1	0	0	0	0	0	1		0		
380	379	0	0	0	0	0	0	0	0	0	0	1		0	0	0
381	380	0		0	0	1	0	0	0	1	0		1			
382	381	0	0	0	0	1	0	0	0	1	0		1			
383	382	0		1	0	0	1	0	0	0	0	1		0		1
384	383	0	0	0	0	1	0	0	0	1	0		1			
385	384	1	1	1	1	0	0	0	0	2	0		1			
386	385	0	0	0	1	1	0	0	0	2	0		1			
387	386	0	0	0	0	0	1	0	0	2	0		1			
388	387	0	0	0	0	0	1	0	0	2	0		1			
389	388	0	0	0	0	1	0	0	0	0	0	1		0	0	0
390	389	0	0	0	0	1	0	0	0	0	0	1		0	0	0
391	390	0	0	0	0	0	1	1	0	0	0	1		0	0	0
392	391	0	1	1	0	1	0	0	0	0	0	1		0	1	1
393	392	0	0	0	0	1	0	0	0	0	0	1		0	0	0
394	393	0	0	0	0	1	0	0	0	0	0	1		0	0	0
395	394	0	0	1	1	1	0	1	0	0	0	1		0	0	1
396	395	0	0	0	0	1	0	0	0	0	0	1		0	0	0
397	396	0	0	0	0	1	0	0	0	0	0	1		0	0	0
398	397	0	0	0	0	1	0	0	0	0	0	1		0	0	0
399	398	0	0	0	0	0	0	0	0	0	0	1		0	0	0
400	399	0	0	0	0	0	0	0	0	0	0	1		0	0	0
401	400	0	1	1	1	0	0	1	0	0	0	1		0	1	1
402	401	0	0	0	0	0	0	0	0	0	0	1		0	0	0
403	402	0	0	0	0	0	0	1	0	0	0	1		0	0	0
404	403	0	0	0	0	1	1	0	0	0	0	1		0	0	0
405	404	0	0	0	0	0	1	0	0	0	0	1		0	0	0
406	405	0	0	0	0	1	0	0	0	0	0	1		0	0	0
407	406	0	0			0	1	0	0	0	0	1		0	0	
408	407	0	0	0	0	0	1	1	0	0	0	1		0	0	0
409	408	0	0	0	0	0	0	0	0	0	0	1		0	0	0
410	409	0	0		0	0	0	0	0	0	0	1		0	0	
411	410	0	0	0	0	1	0	1	0	0	0	1		0	0	0
412	411	0	0	0	0	1	0	1	0	0	0	1		0	0	0
413	412	0	0	0	0	1	0	0	0	0	0	1		0	0	0
414	413	0	1	0	0	1	0	1	0	0	0	1		0	1	0
415	414	0	0	0	0	1	0	0	0	0	0	1		0	0	0
416	415	1	1	1	1	0	0	1	0	1	0		1			
417	416	0	1	0	0	0	0	0	0	0	0	1		0	1	0
418	417	0	0	0	0	0	0	1	0	0	0	1		0	0	0
419	418	0	0	0	0	1	0	0	0	0	0	1		0	0	0
420	419	0			0	0	0	0	0	0	0	1		0		
421	420	0	0	0	0	1	0	0	0	0	0	1		0	0	0
422	421	0	0	0	0	0	0	0	0	0	0	1		0	0	0
423	422	0	0	0	0	0	0	0	0	0	0	1		0	0	0
424	423	0	0	0	0	0	0	1	0	0	0	1		0	0	0
425	424	0	0	1	0	0	0	1	0	1	0		1			
426	425	0	0	0	0	1	0	0	0	0	0	1		0	0	0
427	426	0	0	0	0	1	0	0	0	0	0	1		0	0	0
428	427	0	0	0	0	1	0	1	0	0	0	1		0	0	0
429	428	0	0	0	0	1	0	0	0	0	0	1		0	0	0
430	429	0				0	0	0	0	0	0	1		0		
431	430	0	1	1	1	0	0	0	0	0	0	1		0	1	1
432	431	0	0	0	0	1	0	0	0	0	0	1		0	0	0
433	432	0	0	0	0	0	0	1	0	0	0	1		0	0	0
434	433	0	0	0	0	0	1	1	1	1	0		1			
435	434	0	1	1	1	1	0	0	0	0	0	1		0	1	1
436	435	0	0	0	0	1	0	1	0	0	0	1		0	0	0
437	436	0	0			1	0	0	0	0	0	1		0	0	
438	437	0	1	1	1	1	0	0	0	0	0	1		0	1	1
439	438	0	0	0	0	0	1	1	0	0	0	1		0	0	0
440	439	0	1	1	1	1	0	0	0	0	0	1		0	1	1
441	440	0	0	0	0	0	0	0	0	0	0	1		0	0	0
442	441	0	1	1	1	1	0	1	0	0	0	1		0	1	1
443	442	0	0	0	0	1	0	1	0	0	0	1		0	0	0
444	443	0	0	0	0	0	1	1	0	0	0	1		0	0	0
445	444	0				0	0	0	0	0	0	1		0		
446	445	0	0	0	0	1	0	1	0	0	0	1		0	0	0
447	446	0	0	0	0	1	0	0	0	0	0	1		0	0	0
448	447	0	0	0	0	1	0	0	0	0	0	1		0	0	0
449	448	0	1			0	1	0	0	0	0	1		0	1	
450	449	0	0	0	0	0	0	0	0	0	0	1		0	0	0
451	450	0	0	0	0	1	0	0	0	0	0	1		0	0	0
452	451	0	0	0	0	1	0	0	0	0	0	1		0	0	0
453	452	0	0	0	0	0	0	1	0	0	0	1		0	0	0
454	453	1	0	0	0	1	1	0	0	0	0	1		1	0	0
455	454	0	0		0	1	0	0	0	0	0	1		0	0	
456	455	0	0	0	0	1	0	0	0	2	0		1			
457	456	0	1	1	0	1	0	1	0	2	0		1			
458	457	0				1	0	1	0	2	0		1			
459	458	0	1	0	0	1	0	1	0	2	0		1			
460	459	0	0	1	0	1	0	1	0	2	0		1			
461	460	0	0	0	0	1	0	1	0	2	0		1			
462	461	1	1	0	0	1	0	0	0	2	0		1			
463	462	0	0	0	0	1	0	1	0	2	0		1			
464	463	0	0	0	0	1	0	0	0	1	0		1			
465	464	0	0	0	0	0	0	0	0	1	0		1			

466	465	0	0	0	0	0	1	0	0	1	0		1			
467	466	0	0	0	0	0	1	0	0	1	0		1			
468	467	0	0	0	0	1	0	0	0	1	0		1			
469	468	0	0	0	0	0	1	0	0	2	0		1			
470	469	0	0	0	0	0	0	0	0	0	0	1		0	0	0
471	470	0	0	0	0	0	1	1	0	1	0		1			
472	471	0	0	0	0	0	0	1	0	0	0	1		0	0	0
473	472	0	0	0	0	0	0	1	0	0	0	1		0	0	0
474	473	0	1	0	0	0	1	1	0	2	0		1			
475	474	0	0	0	0	0	1	0	0	1	0		1			
476	475	1	1	0	1	1	0	1	0	2	0		1			
477	476	0	1	1	1	1	0	0	0	1	0		1			
478	477	0	0	0	0	1	1	0	0	0	0	1		0	0	0
479	478	0	0	0	0	0	1	1	0	0	0	1		0	0	0
480	479	0	0	0	0	0	0	1	0	2	0		1			
481	480	0	0	1	1	1	1	0	0	1	0		1			
482	481	0	0	0	0	1	1	1	0	1	0		1			
483	482	0	0	0	0	1	0	1	0	2	0		1			
484	483	0	0	0	0	0	0	1	0	2	0		1			
485	484	0	0	0	0	1	0	1	0	1	0		1			
486	485	0	1	0	0	0	0	1	0	2	0		1			
487	486	0	0	0	0	1	0	1	0	2	0		1			
488	487	0	0	0	0	1	0	1	0	2	0		1			
489	488	0	0	0	0	1	0	1	0	2	0		1			
490	489	0	0	0	0	0	0	1	0	2	0		1			
491	490	0	0	0	0	1	0	1	0	2	0		1			
492	491	0	0		0	1	0	1	0	2	0		1			
493	492	1	1	1	1	1	0	1	0	2	0		1			
494	493	0	0	0	0	0	1	1	1	1	1		1			
